# Dimensions of Parenting and Children’s Conduct Problems: The Importance of Considering Children’s Callous–Unemotional Traits

**DOI:** 10.3390/ijerph21060779

**Published:** 2024-06-14

**Authors:** Carolina Facci, Andrea Baroncelli, Paul J. Frick, Enrica Ciucci

**Affiliations:** 1 Department of Education, Languages, Interculture, Literatures and Psychology, University of Florence, Via di San Salvi 12, Complesso di San Salvi Padiglione 26, 50135 Florence, Italy; carolina.facci@unifi.it (C.F.); enrica.ciucci@unifi.it (E.C.); 2 Department of Philosophy, Social Sciences and Education, University of Perugia, Piazza Giuseppe Ermini 1, 06123 Perugia, Italy; 3 Department of Psychology, Louisiana State University, 208 Audubon Hall, Baton Rouge, LA 70803, USA; pfrick@lsu.edu

**Keywords:** parenting, callous–unemotional traits, conduct problems

## Abstract

Research has clearly indicated that the development of serious behavioral problems in children and adolescents is influenced by parenting. However, recent research has refined the role of parenting by showing the importance of distinguishing between different types of parenting and in considering the role of callous–unemotional traits (CU traits) and conduct problems (CP) of the children. In the current study, we advance this research by distinguishing between emotional (e.g., parental warmth; parental hostility) and behavioral (e.g., use of positive reinforcement; inconsistent discipline/harsh discipline) aspects of parenting and by considering the way parents respond to children’s emotions (i.e., coaching and dismissing). The sample consisted of 136 mothers (M = 38.09 years, SD = 4.51 years, 45.41% high school degree) with a child (age range 3–5 years) enrolled in kindergarten in central Italy. Multiple regression analyses indicated that, after controlling for level of CP, use of positive reinforcement (β = −0.31, *p* < 0.001) and warm feelings (β = −0.22, *p* < 0.05), remained associated with CU traits and punitive parenting was no longer significant. Consistent with predictions, use of positive reinforcement was no longer associated with conduct problems when controlling for CU traits and the positive associations with punitive parenting (β = 0.24, *p* < 0.05) and negativity (β = 0.36, *p* < 0.001) remained significant. These findings support the need for continued research that considers both the emotional and behavioral aspects of parenting and disentangles their associations with conduct problems and CU traits. Such research could not only advance causal theories for children with conduct problems but also help to guide more effective treatments, especially for those with elevated CU traits who often leave treatment with significant conduct problems remaining.

## 1. Introduction

Parenting is critical to most major theories of how children and adolescents develop serious conduct problems [1]. Further, changing parents’ parenting behaviors has been a crucial component of the most effective interventions for preventing and treating conduct problems in young children [2]. Recently, there has been an increasing focus on the role that children’s callous–unemotional (CU) traits may play in both theories for how parenting may be related to conduct problems [3] and how to best intervene in the parent–child relationship for children with behavioral problems [4]. CU traits are defined largely by deficits in prosocial emotions (i.e., lack of remorse or guilt, callousness or lack of empathy, unconcern about performance in important activities, and shallow or constricted affect [5]). Research has shown that children and adolescents with serious conduct problems who also show elevated CU traits exhibit different genetic, cognitive, emotional, personality, and social characteristics compared to those who show normative levels of CU traits [6] and those with elevated CU traits tend to enter treatment with more severe behavior problems and, despite improving with treatment, they often leave traditional mental health treatments with more severe behavior problems [7]. As a result of this research, CU traits have recently been included as a specifier for mental health diagnoses for children and adolescents with severe behavior problems in the major classification systems used worldwide [8,9].

Research has suggested that the associations between parenting and conduct problems may differ depending on the presence of CU traits and these differences may play a role in the reduced effectiveness of interventions for children with these traits. Specifically, several studies have suggested that harsh and inconsistent parenting may be more related to conduct problems in those who do not show elevated CU traits, whereas warm and responsive parenting may be more related to conduct problems in children and adolescents elevated on CU traits [10,11,12]. Further, most parenting interventions used to treat conduct problems in young children often focus largely on reducing harsh and inconsistent parenting, with less attention paid to increasing parental warmth [2]. However, when parenting interventions are modified to include a greater focus on enhancing parental warmth, they have resulted in better outcomes for children with elevated CU trait [4,13].

Based on this research, it is critical to consider harsh/inconsistent parenting and warm/responsive parenting separately and to consider the separate effects of each of these dimensions on conduct problems and CU traits (see [14]). However, even this distinction between negative and positive parenting may not be specific enough to detect important differences in their associations with conduct problems and CU traits. That is, each of these dimensions can include both parenting feelings towards their child (i.e., hostile vs. warm feelings), as well as parental behaviors (i.e., inconsistent/harsh discipline vs. use of positive reinforcement). Developmental research has suggested that the emotional climate provided by the parent and the parenting behaviors can have separate effects on child development [15]. Specifically related to CU traits, Clark and Frick [16], reported that, in a sample of kindergarten students, parental use of positive reinforcement was more strongly (i.e., negatively) related to conduct problems in those high on CU traits, whereas warm feelings towards their child was more related to the CU traits themselves. These findings would be consistent with developmental theories suggesting that children develop prosocial emotions through a warm parent–child relationship, in which they learn how to recognize emotions in others and are motivated to be responsive to the feelings of others through this warm and mutually cooperative relationship between the parent and child [17]. Unfortunately, only limited research to date has systematically separated the emotional and behavioral aspects of parenting in testing their associations with conduct problems and CU traits.

Another potentially important avenue for advancing this important area of research is focusing on how parents respond to emotions in their child. That is, most parenting measures focus on how parents respond to the child’s behavior (e.g., [18]). However, developmental research has suggested that how parents respond to a child’s emotions could be critical for their development of appropriate levels of emotional regulation. That is, parent responses that encourage appropriate emotional expression (i.e., emotional coaching) result in children developing better emotional regulation and more appropriate prosocial emotions towards others, as compared to parents who discourage emotional expression (i.e., dismissive responses) [19]. Further, another way that parenting interventions have been modified to be more effective for children with elevated CU traits has been to add a component that helps parents learn and practice emotional coaching techniques, in addition to other changes in their expression of emotions towards and use of discipline with their child [4,13]. Unfortunately, to date, parental responses to emotions have not been consistently studied in relation to CU traits and conduct problems, which could be important for advancing both causal theories on the development of conduct problems and for enhancing parenting interventions even further for children with elevated CU traits.

### The Current Study

Based on this research, the current study provides a further investigation into the associations between various dimensions of parenting and conduct problems in children. Based on extensive past research, we considered the potential role of CU traits in this association. That is, based on past research (e.g., [14]), we predicated that positive dimensions of parenting would be more related to conduct problems in those elevated on the CU traits scale, whereas harsh and inconsistent parenting would be more related to conduct problems in those lower on the CU traits scale. Further based on past research [16], we predicated that within the positive dimension of parenting, parental use of positive reinforcement would be more related (negatively) to conduct problems in those elevated on the CU traits scale, whereas warm feelings towards the child would be more related to the CU traits themselves (again, negatively). Finally, we tested a novel hypothesis that the parental use of an emotional coaching style would be negatively related to CU traits, whereas the use of dismissive reactions to emotions would be positively related to these traits.

## 2. Materials and Methods

### 2.1. Participants and Procedures

Participants (n = 163) were recruited from kindergartens in central Italy (October-November/2021). Children ranged in age from 3 to 5 years (41.7% attending the class of the “youngest”—3 years old) that for the Italian educational system is the age range at which children can attend kindergartens. Mothers ranged in age from 26 to 49 years (M = 38.09, SD = 4.51). In terms of education, only 6.71% of the sample reported have less than a high school education, 45.41% have a high school degree, 35,01% have a college degree, and 12.87% have also attended post-graduate courses. Most mothers reported being Italian (91.87%).

Three scholastic institutions approved and collaborated with all procedures. Mothers were presented with a description of the study that was developed in collaboration with scholastic institutions and teachers, followed by a request for informed consent to participate and to then complete an internet survey about their child, with demographic information and details about the parents (e.g., school degree, family composition). Study measures were presented in a Google Form survey session, with no time restriction to complete the survey.

### 2.2. Measures

#### 2.2.1. Parenting Behaviors

The Alabama Parenting Questionnaire (APQ; [18]) preschool version (APQ-Pr; [20]) is a 32 item measure developed by taking the subset of items that are relevant for young children. Consistent with past uses of the APQ-Pr [20], we used the 7 item Inconsistent Parenting subscale, which measures a lack of follow-through with discipline (e.g., “You threaten to punish your child and then do not actually punish him/her”), the 5 item Punitive Parenting subscale, which assesses how often a parent engages in corporal or harsh discipline (e.g., “You spank your child with your hand when he/she has done something wrong”), and the 12 Positive Parenting subscale, which assesses parental use of positive reinforcement and parental involvement (e.g., “You let your child know when he/she is doing a good job with something”). In the present study, we used the Italian version of the APQ-Pr [21]. The internal consistencies for the current sample were acceptable for the Positive Parenting scale (α = 0.76) and modest for the Inconsistent Parenting scale (α = 0.67) and Punitive Parenting scale (α = 0.60).

#### 2.2.2. Maternal Emotional Style

The Maternal Emotional Styles Questionnaire (MESQ; [22]) contains 14 items assessing maternal emotional coaching (EC) and maternal emotional dismissing (ED) parenting styles using 7 items each. Mothers are asked to express their level of agreement with each of the 14 items, on a 5 point Likert scale ranging from 1 (strongly disagree) to 5 (strongly agree). The items assess the degree to which the mother agrees with different ways of responding to various emotions in their child. For example, a sample EC item is “When my child is angry, I take some time to experience this feeling with him/her” and a sample ED item is “When my child is angry, my goal is to make him/her stop”. The MESQ was translated into Italian by Ciucci and Menesini [23].

We ran an exploratory factor analysis (EFA) with the IBM SPSS Statistics 28 program [24] adopting the principal axis method and a Promax rotation. The results from our dataset indicated an acceptable KMO index of 0.80 and Bartlett’s sphericity test was χ^2^ = 612.04, df = 91, *p* < 0.001. There were two factors with eigenvalues greater than 1.00 and they explained 45.33% of the variance in the items. An item (“When my child is angry, I want to know what he/she is thinking”) failed to load (i.e., a factor loading greater than |.40|, with a gap > |.10| between the two factor loadings) on any factor and was excluded from subsequent analyses. Another item (“When my child is angry, it’s time to solve his/her problem”) reached the retention criteria but it was not considered in the subsequent analyses because of a factor loading that was inconsistent with the theory guiding scale development (i.e., loading on coaching instead of dismissing), probably due to the Italian translation. To summarize, the first factor accounted for 27.50% of the variance in the items and its rotated factor loadings ranged from 0.42 to 0.69. The items loading on this factor were the 7 items reflecting the ED parenting style and the Cronbach’s alpha for this subscale was 0.81. The second factor accounted for 17.83% of the variance in items and the rotated factor loadings ranged from 0.47 to 0.61. The 5 items on this factor were items reflecting the EC parenting style and the Cronbach’s alpha was 0.66 for this subscale.

#### 2.2.3. Parental Feelings

The Parent Feelings Questionnaire (PFQ; [25]) is a 24 item measure that assesses both positive and negative parental feelings toward their children and it is widely used in preschool samples [26]. The measure includes statements about feelings that are rated on a 5 point Likert-type scale ranging from 1 (completely agree) to 5 (completely disagree). The measure includes two subscales; the Parental Warmth subscale comprises 11 items assessing positive parental feelings (e.g., “When I think about this child, it usually gives me warm feelings”) and the Negativity subscale comprises 13 items assessing negative parental feelings (e.g., “Sometimes I am not happy about my relationship with this child”).

An EFA of these 24 items resulted in an acceptable KMO index of 0.84 and the Bartlett’s sphericity test was χ^2^ = 1403.01, df = 276, *p* < 0.001. Consistent with past uses of the scale, two factors emerged with eigenvalues greater than 1.00 and they explained 37.84% of the variance in the item sets. However, 4 items resulted in factor loadings lower than 0.40 and were excluded from subsequent analyses. Factor 1 included the 14 items reflecting the negative feelings “negativity”. It accounted for 28.10% of the variance in the items and its factor loadings ranged from 0.44 to 0.74. The Cronbach’s alpha for this subscale was 0.90. Factor 2 included 6 items related to “warm feelings” and it accounted for 9.73% of the variance in the items. Its factor loadings ranged from 0.42 to 0.63 and resulted in a Cronbach’s alpha of 0.60.

#### 2.2.4. Callous–Unemotional Traits

To measure callous–unemotional traits, we used the parent report version of the Inventory of Callous–Unemotional traits (ICU, [27]), that was translated into Italian and validated by Ciucci and colleagues [28]. We used the preschool version of the ICU, a 24-item scale (e.g., “my child is concerned about the feelings of others”—reversed) that slightly modifies two items to be more appropriate for young children [29]. The Cronbach’s alpha in the present sample was 0.80 for the ICU total score.

#### 2.2.5. Conduct Problems

Conduct problems were measured using the conduct problems subscale of the Strengths and Difficulties Questionnaire (SDQ [30], which was translated and validated in Italian by Tobia and Marzocchi [31]. The SDQ is one of the most widely used measures of child emotional and behavioral adjustment. The 5 item conduct problem scale was used in the current study (e.g., “Often lies or cheats”). Each item is rated on a three-point Likert scale ranging from 0 = “not true” to 2 = “certainly true”. Cronbach’s alpha of this subscale in the current sample was 0.60.

### 2.3. Data Analyses

All the analyses were conducted using SPSS version 28 [24]. Prior to testing the main study hypotheses, descriptive statistics and bivariate correlations among the main study variables were computed. Then, to test the unique and interactive associations with CU traits and CP, we ran seven hierarchical multiple regression analyses with each parenting measure as a dependent variable. CU traits were entered in the first step and then CP was added at step 2. In a third step, we added the interaction between CU traits and CP to determine if any interaction modified the main effects of these two variables. Before performing multiple regression analysis, all the continuous variables were centered by subtracting the sample mean from scores. We also tested the possible role of maternal age, maternal education, and child school grade and we found no significant correlations with the study variables; for this reason, we did not include them in the main analyses.

## 3. Results

The distribution of main study variables and zero-order correlations among the main study variables are provided in Table 1. As expected, there were significant positive correlations between CU traits and conduct problems (r = 0.49, *p* < 0.001). In addition, there were modest-to-moderate correlations between the parenting variables and both CU traits and conduct problems. Specifically, both CU traits and conduct problems were positively associated with positive parenting and warmth feelings and were negatively correlated with inconsistent parenting, punitive parenting, and negativity. None of the correlations testing the associations between emotional coaching and dismissing parenting were significantly correlated with either CU traits or conduct problems.

The results of the hierarchical multiple regression analyses are provided in Table 2. Consistent with predictions, positive parenting (β = −0.31, *p* < 0.001) and warmth feelings (β = −0.22, *p* < 0.05) remained associated with CU traits and the association between CU traits and punitive parenting was no longer significant, after controlling for level of conduct problems. However, contrary to predictions, inconsistent parenting (β = 0.21, *p* < 0.05) and negativity (β = 0.19, *p* < 0.05) remained significantly associated with CU traits after controlling for conduct problems. Consistent with predictions, positive parenting was no longer associated with conduct problems when controlling for CU traits and the positive associations with punitive parenting (β = 0.24, *p* < 0.05) and negativity (β = 0.36, *p* < 0.001) remained significant. However, contrary to predictions, parental warmth (β = −0.18, *p* < 0.05) remained significantly associated with conduct problems after controlling for CU traits.

None of the significant interactions between CU traits and conduct problems reached statistical significance.

## 4. Discussion

The present study investigated the associations between various dimensions of parenting and conduct problems in children, while considering the potential role of CU traits. These tests were done within an Italian sample of mothers with children aged 3 to 5 years. To advance past research, our analyses not only made a distinction between positive and negative aspects of parenting, but also distinguished between parental feelings about their child (e.g., warm vs harsh) and parenting behaviors designed to socialize their child (e.g., use of positive reinforcement and use of harsh and inconsistent parenting techniques). Showing the importance of considering both the shared and independent associations of parenting dimensions with conduct problems and CU traits [15], both the positive and negative aspects of parenting and both parenting emotions and parenting behaviors were significantly associated with CU traits and conduct problems in zero-order correlations. However, some differential associations emerged in multiple regression analyses testing the unique associations with each child characteristic.

Specifically, when controlling for conduct problems, positive parenting behaviors (i.e., parental involvement and use of positive reinforcement) were uniquely associated with CU traits. These results would be consistent with research suggesting that children with CU traits may be more sensitive to rewards than punishments [6] and this motivational set may be why children with CU traits respond well to interventions that focus on helping parents increase their use of positive reinforcement [13,14]. In contrast, when controlling for the level of CU traits, use of harsh discipline was uniquely associated with conduct problems. While our study was cross-sectional, the results are consistent with a longitudinal study by Goulter and colleagues [32] who reported that parental harsh punishment assessed in grades 1 and 2 was related to conduct problems but not CU traits in grade 6. Although not tested in either the current study or by Goulter and colleagues [32], it is highly likely that this association between harsh parenting and conduct problems is bidirectional, with a child’s conduct problems evoking more harsh parenting and the harsh parenting leading to more conduct problems in an escalating coercive cycle [33]. Also, this finding that harsh parenting is more related to conduct problems than CU traits is consistent with theories specifying that youth with CU traits who experience harsh discipline are more likely to exhibit higher rates of both emotional (i.e., anxiety) and behavioral (i.e., conduct problems and aggression) problems [34].

Interestingly, and counter to our predictions, inconsistent parenting was significantly related to CU traits but not conduct problems in multiple regression analyses. This unexpected finding may be due to separating harsh and inconsistent parenting behaviors into separate dimensions and may explain why past research using measures that combine harsh and inconsistent parenting have resulted in inconsistent findings. For example, Waller and colleagues [35] reported that, in a sample of 227 monozygotic twin pairs (ages 6 to 11 years), a measure that combined parental inconsistency and harshness was related to both aggression and CU traits.

Importantly, our findings using measures of parental feelings towards their child led to a very different pattern of results than was found for parenting behaviors. That is, while parenting negative feelings toward the child were somewhat more highly associated with conduct problems than CU traits as predicted, both positive and negative parenting feelings towards their child remained significantly associated with both CU traits and conduct problems in multiple regression analyses. While these results were unexpected, they do support the need to separate parenting behaviors and parenting emotions towards their child in future research [15,16]. Further, they support the importance of parental warmth as a significant target of intervention for children with elevated CU traits, both for decreasing their conduct problems and for decreasing their level of CU traits [4,13]. Again, while our results are cross-sectional and cannot address the directionality of the associations between parental feelings and child outcomes, past research testing the bidirectional associations using strong longitudinal methods have suggested that CU traits seem to lead to decreases in parental warmth over time [36]. Our results suggest that CU traits may also lead to an increase in parental negative feelings towards the child, even when controlling for the child’s level of conduct problems.

Another unique contribution of the current study was to test whether parental responses to children’s emotions were differentially related to conduct problems and CU traits. This was important because, while parental coaching (as opposed to dismissing) of their children’s display of emotions has been shown to increase appropriate emotional regulation in young children [20], most measures of parenting that have been used in past research in relation to child conduct problems have largely focused on parents’ responses to their children’s behavior and not their responses to their children’s emotions. However, our measure of emotional coaching was not related to either CU traits or conduct problems. One possible reason for this finding is that most studies of parental emotional coaching used in developmental research involved observations of parent–child interactions and recorded parents’ responses to their children’s emotional display [20], whereas we relied on parental reports of their emotional coaching behaviors. Thus, it may be that parents are not good reporters of the strategies that they use in response to their children’s emotions. Alternatively, it could be that our emotional coaching measure measured parental responses to various types of emotions. There is evidence that children with elevated CU traits may have very specific deficits in the types of emotions for which they may have difficulty processing [37]. Thus, a measure that allowed for a differentiation in parental responses across various types of emotions may have led to significant associations. 

Finally, a finding that was not consistent with predictions was the absence of any interactions between CU traits and conduct problems in their association with the parenting measure. Of note, most studies that have found such interactions have been conducted in clinic-referred [10,11] or detained [38] samples. Further, in most samples, CU traits and conduct problems do not show bivariate normality, in that there are significant numbers of youth high on conduct problems with both high and low levels of CU traits. However, there typically are few youths who score high on CU traits but are low on conduct problems. This asymmetrical overlap in distributions, which is typically more apparent in community samples, is why interaction effects may not be found in non-referred samples [5].

These findings should be interpreted in the context of several study limitations. First, as noted above, the cross-sectional design of the study did not allow us to assess bidirectional associations between our parenting measures and child characteristics or to make causal inferences from our results. Second, both the size and representativeness of our sample limit the generalizability of results. The sample used in this study was primarily Italian, from central regions and was of all mothers. Different cultural perspectives and differences across mothers and fathers may affect how parents think about emotions, and specifically about their children’s emotions [39]. Another important aspect that should be considered in future research would be the income and education level of the families that may also relate to how parents interact with their children [40]. Therefore, additional work is needed to determine how well our results may generalize to different parenting contexts. Third, as noted above, we relied on parental reports for our measure of emotional coaching, which may have led to our failure to find associations with this measure. However, it is important to note that we relied on parental reports for all of our measures of parenting dimensions and for all of our measures of children’s characteristics. As a result, our measures are subject to potential biases and subjectivity in the parents’ reports, and our correlations between parenting and child characteristics could be inflated due to shared method variance. However, it is not likely that such method variance would influence the associations with one child characteristic (e.g., CU traits) more than the other (e.g., conduct problems).

## 5. Conclusions

The findings support the need to consider the role of CU traits when studying the associations between parenting and conduct problems. That is, harsh parenting appeared to be more specifically related to conduct problems, while the use of positive reinforcement appeared to be uniquely associated with CU traits. These findings could be critical, not just for explaining how these two related child characteristics develop but also for guiding interventions to address them. Most interventions for conduct problems focus more on reducing harsh parenting than on increasing parental use of positive reinforcement and this may be why children with elevated CU traits end these treatments with more severe conduct problems [7]. This finding may also be why modified interventions that provide enhanced guidance to parents on the use of positive reinforcement may be more effective for these children [4,13]. Moreover, our results suggest that studying these dimensions in preschool children is critically important considering that CU traits and conduct problems were already highly correlated and that the parent–child relationship is quite challenging during this developmental period. Further, our results also support the need to distinguish between parental feelings towards their child and their parenting behaviors. Our findings support the association between CU traits and parental warmth, possibly as an indicator of the effects of raising a child with CU traits on the parent–child relationship [36]. However, our findings suggest that the child’s conduct problems may contribute to these relationships difficulties and that CU traits are also related to higher levels of parental negative feelings towards their children. Possibly interventions towards parents should consider both raising awareness about the affective dimensions towards the children as well as promoting emotional competence in them, since sensitive parental responses to children’s emotions and parental warmth are critical for conscience development during childhood age. Future research using longitudinal designs needs to further explore potential bidirectional influences between a parent’s socialization efforts and the child’s influence on the parent–child relationship. While we have focused on the parent–child relationship, this could also have implications for other socializing agents. Specifically, educators and teachers also play important roles in the emotional socialization of the child [40,41] and similar attention should be devoted to understanding the critical dimensions that make teachers more or less effective in this role and how a child’s characteristics may influence these socialization efforts.

## Figures and Tables

**Table 1:** Distribution of main study variables and zero-order correlations (Pearson’s *r*).

Variable	M	SD	Skew	Kurt	1	2	3	4	5	6	7	8	9
1 ICU Pr–total score	0.65	0.33	0.46	−0.25	/	0.49 ***	−0.33 ***	0.23 **	0.16 *	−0.31 ***	0.35 ***	−0.06	−0.05
2 SDQ–Conduct Problems	1.33	0.31	0.95	0.57		/	−0.18 *	0.22 **	0.26 ***	−0.29 ***	0.46 ***	−0.11	−0.07
3 APQ–Positive Parenting	4.53	0.35	−0.92	0.57			/	−0.01	−0.04	0.33 ***	−0.35 ***	0.16 *	0.20 *
4 APQ–Inconsistent Parenting	2.55	0.71	0.11	−0.31				/	0.16 *	−0.20 **	0.22 **	−0.10	0.39 ***
5 APQ–Punitive Parenting	2.17	0.63	0.60	0.52					/	−0.43 ***	0.34 ***	−0.11	0.16 *
6 PFQ–Warmth feelings	4.68	0.39	−1.90	4.02						/	−0.34 ***	0.28 ***	−0.10
7 PFQ–Negativity	2.31	0.80	0.40	−0.61							/	−0.10	−0.18 *
8 MESQ–Coaching	4.43	0.58	−1.19	1.50								/	−0.06
9 MESQ–Dismissing	3.30	0.82	0.03	−0.67									/

Notes: ICU Pr: Inventory of Callous–Unemotional Traits Preschool Version; SDQ: Strengths and Difficulties Questionnaire, MESQ: Maternal Emotional Style Questionnaire; PFQ: Parent Feeling Questionnaire; APQ: Alabama Parenting Questionnaire. * *p* < 0.05, ** *p* < 0.01, *** *p* < 0.001.

**Table 2:** Hierarchical Regression Analyses on parenting variables.

	APQ–Positive Parenting	APQ–Inconsistent Parenting	APQ–Punitive Parenting	PFQ–Warmth Feelings	PFQ–Negativity	MESQ–Coaching	MESQ–Dismissing
**Step 1**	F(1, 161) = 19.062 *** R^2^= 0.10	F(1, 161) = 12.387 *** R^2^ = 0.07	F (1, 161) = 4.387 * R^2^= 0.02	F (1, 161) = 16.729 *** R^2^= 0.09	F (1, 161) = 25.165 *** R^2^= 0.13	F (1, 161) = 0.959 R^2^= 0.00	F (1, 161) = 0.630 R^2^ = 0.00
	*β*	*β*	*β*	*β*	*β*	*β*	*β*
ICU-Pr	−0.33 ***	0.27 ***	0.16 *	−0.31 ***	0.37 ***	−0.07	−0.06
**Step 2**	F(2, 161) = 9.541 *** ΔR^2^ = 0.01; R^2^ = 0.09	F(2, 161) = 7.041 *** ΔR^2^ = 0.00; R^2^ = 0.07	F(2, 161) = 6.098 ** ΔR^2^= 0.04 **; R^2^= 0.06	F(2, 161) = 10.785 ΔR^2^ = 0.02 *; R^2^ = 0.11	F(2, 161) = 24.662 ΔR^2^ = 0.10 ***; R^2^ = 0.23	F(2, 161) = 1.052 ΔR^2^ = 0.00; R^2^ = 0.00	F(2, 161) = 0.519 ΔR^2^ = 0.00; R^2^ = 0.00
	*β*	*β*	*β*	*β*	*β*	*β*	*β*
ICU-Pr	−0.31 ***	0.21 *	0.04	−0.22 *	0.19 *	−0.03	−0.03
SDQ-CP	−0.03	0.11	0.24 **	−0.18 *	0.36 ***	−0.10	−0.06

Notes: β = standardized beta coefficient; SDQ = Strengths and Difficulties Questionnaire; CP = conduct problems; MESQ: Maternal Emotional Style Questionnaire; PFQ: Parent Feeling Questionnaire; APQ: Alabama Parenting Questionnaire. * *p* < 0.05; ** *p* < 0.01; *** *p* < 0.001.

## Data Availability

There are no unpublished data available. The corresponding author can be contacted regarding this matter.

## References

[1] Frick P.J., Viding E. (2009). Antisocial behavior from a developmental psychopathology perspective. Dev. Psychopathol..

[2] McMahon R.J., Frick P.J., Prinstein M.J., Youngstrom E.A., Mash E.J., Barkley R.A. (2019). Conduct and oppositional disorders. Treatment of Disorders in Childhood and Adolescence.

[3] Waller R., Gardner F., Hyde L.W. (2013). What are the associations between parenting, callous-unemotional traits, and antisocial behavior in youth? A systematic review of evidence. Clin. Psychol. Rev..

[4] Kimonis E.R., Fleming G., Briggs N., Brouwer-French L., Frick P.J., Hawes D.J., Bagner D.M., Thomas R., Dadds M. (2019). Parent-child interaction therapy adapted for preschoolers with callous-unemotional traits: An open trial pilot study. J. Clin. Child Adolesc. Psychol..

[5] Frick P.J. (2022). Some critical considerations in applying the construct of psychopathy to research and classification of childhood disruptive behavior disorders. Clin. Psychol. Rev..

[6] Frick P.J., Ray J.V., Thornton L.C., Kahn R.E. (2014). Annual research review: A developmental psychopathology approach to understanding callous-unemotional traits in children and adolescents with serious conduct problems. J. Child Psychol. Psychiatry.

[7] Perlstein S., Fair M., Hong E., Waller R. (2023). Treatment of childhood disruptive behavior disorders and callous-unemotional traits: A systematic review and two multilevel meta-analyses. J. Child Psychol. Psychiatry.

[8] American Psychiatric Association (2013). Diagnostic and Statistical Manual of Mental Disorders.

[9] World Health Organization (2019). International Statistical Classification of Diseases and Related Health Problems, 11th ed. https://icd.who.int/.

[10] Wootton J.M., Frick P.J., Shelton K.K., Silverthorn P. (1997). Ineffective parenting and childhood conduct problems: The moderating role of callous-unemotional traits. J. Consult. Clin. Psychol..

[11] Pasalich D.S., Dadds M.R., Hawes D.J., Brennan J. (2011). Do callous-unemotional traits moderate the relative importance of parental coercion versus warmth in child conduct problems? An observational study. J. Child Psychol. Psychiatry.

[12] Waller R., Gardner F., Shaw D.S., Dishion T.J., Wilson M.N., Hyde L.W. (2015). Callous-unemotional behavior and early-childhood onset of behavior problems: The role of parental harshness and warmth. J. Clin. Child Adolesc. Psychol..

[13] Fleming G.E., Neo B., Kaouar S., Kimonis E.R. (2023). Treatment outcomes of children with primary versus secondary callous-unemotional traits. Res. Child Adolesc. Psychopathol..

[14] Vaughan E.P., Frick P.J., Robertson E.L., Thornton L.C., Wall Myers T.D., Steinberg L., Cauffman E. (2023). Interpersonal relationships and callous-unemotional traits during adolescence and young adulthood: An investigation of bidirectional effects in parent, peer, and romantic relationships. Clin. Psychol. Sci..

[15] Kochanska G., Boldt L.J., Goffin K.C. (2019). Early relational experience: A foundation for the unfolding dynamics of parent–child socialization. Child Dev. Perspect..

[16] Clark J.E., Frick P.J. (2018). Positive parenting and callous-unemotional traits: Their association with school behavior problems in young children. J. Clin. Child Adolesc. Psychol..

[17] Kochanska G. (1997). Multiple pathways to conscience for children with different temperaments: From toddlerhood to age 5. Dev. Psychol..

[18] Shelton K.K., Frick P.J., Wootton J. (1996). Assessment of parenting practices in families of elementary school-age children. J. Clin. Child Psychol..

[19] Johnson A.M., Hawes D.J., Eisenberg N., Kohlhoff J., Dudeney J. (2017). Emotion socialization and child conduct problems: A comprehensive review and meta-analysis. Clin. Psychol. Rev..

[20] Clerkin S.M., Marks D.J., Policaro K.L., Halperin J.M. (2007). Psychometric properties of the Alabama Parenting Questionnaire—Preschool revision. J. Clin. Child Adolesc. Psychol..

[21] Benedetto L., Ingrassia E.M. (2014). L’Alabama Parenting Questionnaire per la fascia prescolare (APQ-Pr). Contributo all’adattamento italiano. Disturbi Attenzione Iperattività.

[22] Lagacé-Séguin D.G., Coplan R.J. (2005). Maternal emotional styles and child social adjustment: Assessment, correlates, outcomes and goodness of fit in early childhood. Soc. Dev..

[23] Ciucci E., Menesini E., Albanese O., Molina P. (2008). La comprensione delle emozioni nei bambini e lo stile emotivo materno. Lo Sviluppo della Comprensione delle Emozioni e la sua Valutazione.

[24] IBM Corp. (1996). Released 2021. IBM SPSS Statistics for Windows, Version 28.0. Armonk, NY: IBM CorpDeater-Deckard, K. Parent Feelings Questionnaire. J. Abnorm. Psychol..

[25] Denham S.A., Wyatt T.M., Bassett H.H., Echeverria D., Knox S.S. (2009). Assessing social-emotional development in children from a longitudinal perspective. J. Epidemiol. Community Health.

[26] Frick P.J., Ray J.V. (2015). Evaluating callous-unemotional traits as a personality construct. J. Personal..

[27] Ciucci E., Baroncelli A., Franchi M., Golmaryami F.N., Frick P.J. (2014). The association between callouunemotional traits and behavioral and academic adjustment in children: Further validation of the Inventory of Callous-Unemotional Traits. J. Psychopathol. Behav. Assess..

[28] Kimonis E.R., Fanti K.A., Anastassiou-Hadjicharalambous X., Mertan B., Goulter N., Katsimicha E. (2016). Can Callous-Unemotional Traits be Reliably Measured in Preschoolers?. J. Abnorm. Child Psychol..

[29] Goodman R., Meltzer H., Bailey V. (1998). The Strengths and Difficulties Questionnaire: A pilot study on the validity of the self-report version. Eur. Child Adolesc. Psychiatry.

[30] Tobia V., Marzocchi G.M. (2018). The Strengths and Difficulties Questionnaire-Parents for italian school-aged children: Psychometric properties and norms. Child Psychiatry Hum. Dev..

[31] Goulter N., McMahon R.J., Pasalich D.S., Dodge K.A. (2020). Indirect effects of early parenting on adult antisocial outcomes via adolescent conduct disorder symptoms and callous-unemotional traits. J. Clin. Child Adolesc. Psychol..

[32] Smith J.D., Dishion T.J., Shaw D.S., Wilson M.N., Winter C.C., Patterson G.R. (2014). Coercive family process and early-onset conduct problems from age 2 to school entry. Dev. Psychopathol..

[33] Meehan A.J., Maughan B., Cecil C.A.M., Barker E.D. (2017). Interpersonal callousness and co-occurring anxiety: Developmental validity of an adolescent taxonomy. J. Abnorm. Child Psychol..

[34] Waller R., Hyde L.W., Klump K.L., Burt S.A. (2018). Parenting is an environmental predictor of callous-unemotional traits and aggression: A monozygotic twin differences study. J. Am. Acad. Child Adolesc. Psychiatry.

[35] Vaughan E.P., Frick P.J., Ray J.V., Robertson E.L., Thornton L.C., Wall Myers T.D., Steinberg L., Cauffman E. (2021). The associations of maternal warmth and hostility with prosocial and antisocial outcomes in justice-involved adolescents. Dev. Psychol..

[36] Frick P.J., Kemp E.C. (2021). Conduct disorders and empathy development. Annu. Rev. Clin. Psychol..

[37] Edens J.F., Skopp N.A., Cahill M.A. (2008). Psychopathic features moderate the relationship between harsh and inconsistent parental discipline and adolescent antisocial behavior. J. Clin. Child Adolesc. Psychol..

[38] Parker A.E., Halberstadt A.G., Dunsmore J.C., Townley G., Bryant A., Thompson J.A., Beale K.S. (2012). Emotions are a window into one’s heart: A qualitative analysis of parental beliefs about children’s emotions across three ethnic groups. Monogr. Soc. Res. Child Dev..

[39] Taraban L., Shaw D. (2018). Parenting in context: Revisiting Belsky’s classic process of parenting model in early childhood. Dev. Rev..

[40] Baroncelli A., Ciucci E. (2020). Bidirectional effects between callous-unemotional traits and student-teacher relationship quality among middle school students. J. Abnorm. Child Psychol..

[41] Baroncelli A., Facci C., Ciucci E. (2022). Sensitivity to teachers’ punishment and social affiliation with teachers: Unique and interactive effects to callous-unemotional traits among preadolescents. J. Res. Pers..

